# Dysphagia Due to an Extremely Long Styloid Process: A Case Report of Eagle Syndrome

**DOI:** 10.7759/cureus.34250

**Published:** 2023-01-26

**Authors:** Ali Albayat, Ali Al Habeeb, Mahdi Jawad

**Affiliations:** 1 General Practice, Qatif Central Hospital, Qatif, SAU; 2 Radiology, Qatif Central Hospital, Qatif, SAU

**Keywords:** case report, computed tomography, styloid process, eagle's syndrome, neck pain, dysphagia

## Abstract

Dysphagia is a relatively common condition in the general population and has a wide range of underlying etiologies. We present the case of a 58-year-old male who presented with a complaint of progressive difficulty swallowing for two years in duration associated with unintentional weight loss. He has been using a proton pump inhibitor therapy for more than one year, but he had only mild improvement in his symptoms. Recently, the patient started to experience neck pain during swallowing and he underwent a head and neck computed tomography scan, which demonstrated an extensive elongation of the left styloid process that measured 14.9 cm. The clinical and imaging findings were consistent with Eagle syndrome and the decision was made to perform a resection of the left styloid process. Excision of the left styloid process was made using the external cervical approach. At the follow-up visit, the patient reported a near-complete resolution of his complaints. Eagle syndrome is a very rare etiology of dysphagia. The case highlights an example of Eagle syndrome with an extremely long styloid process. This diagnosis should be considered when encountering a patient with dysphagia and neck pain.

## Introduction

Dysphagia is a relatively common complaint in the general population. A population-based survey study involving 30,000 adults found that one in six adults reported a subjective difficulty in swallowing [1]. It is more common in patients with gastroesophageal reflux disease. The styloid process is a slender bone that arises from the petrous part of the temporal bone and serves as an attachment site for the stylohyoid ligament and certain muscles [2]. The styloid process is related to the carotid artery, the internal jugular vein, and multiple nerves such as the vagus, glossopharyngeal, and accessory nerves. The styloid process varies in length, but it is considered abnormal if it is longer than 4 cm and was associated with symptoms. The longest length reported for the styloid process was 14 cm [3]. It is a disease with idiopathic etiology, but several underlying pathophysiological mechanisms have been proposed to cause pain and symptoms of the syndrome. For example, the abnormal styloid process may cause compression of the carotid artery, resulting in syncope [1-3].

Eagle syndrome is a rare clinical condition related to abnormalities related to the styloid process [2]. Such abnormalities include long length, increased inward deviation, and anterior angulation of the styloid process [1-3]. The diagnosis of Eagle syndrome can be challenging because of the non-specific presenting symptoms as it can present in different clinical manifestations (e.g., ear pain, neck pain, and dysphagia) [2]. The first description of this syndrome was made by Watt Eagle in 1937 who provided two different syndromes associated with the prolonged styloid process [4]. The first description was for the syndrome associated with dysphagia and a foreign body sensation, while the second description involved carotid artery compression and its associated symptoms. However, Eagle syndrome has a wide spectrum of manifestations and its clinical diagnosis is challenging [5].

## Case presentation

We present the case of a 58-year-old male who came to our outpatient clinic with a complaint of difficulty swallowing for a period of two years. The patient experienced difficulty in swallowing solid food, which worsened progressively in the past few months as he unintentionally lost around 5 kg over two months. He reported that he felt the food was stuck behind his chest. The patient was seen by multiple physicians who diagnosed him with gastroesophageal reflux disease. He has been using a proton pump inhibitor therapy for more than one year, but he had only mild improvement in his symptoms. There was no history of choking during swallowing or hoarseness. Recently, the patient started to experience neck pain during swallowing. The pain was aggravated by the turning of the head. No radiation to the ears was reported. The past medical history was remarkable for irritable bowel syndrome, hypertension, diabetes mellitus, and asthma. He did not have any operations in the head and neck region. His medications include amlodipine 5 mg, metformin 850 mg, aspirin 75 mg, atorvastatin 10 mg, and inhaled budesonide. He was a heavy smoker with a 50-pack-year smoking history. He never consumed alcohol. The family history was remarkable for colon cancer in two of his siblings that were diagnosed before the age of 50 years.

**Figure 1:**
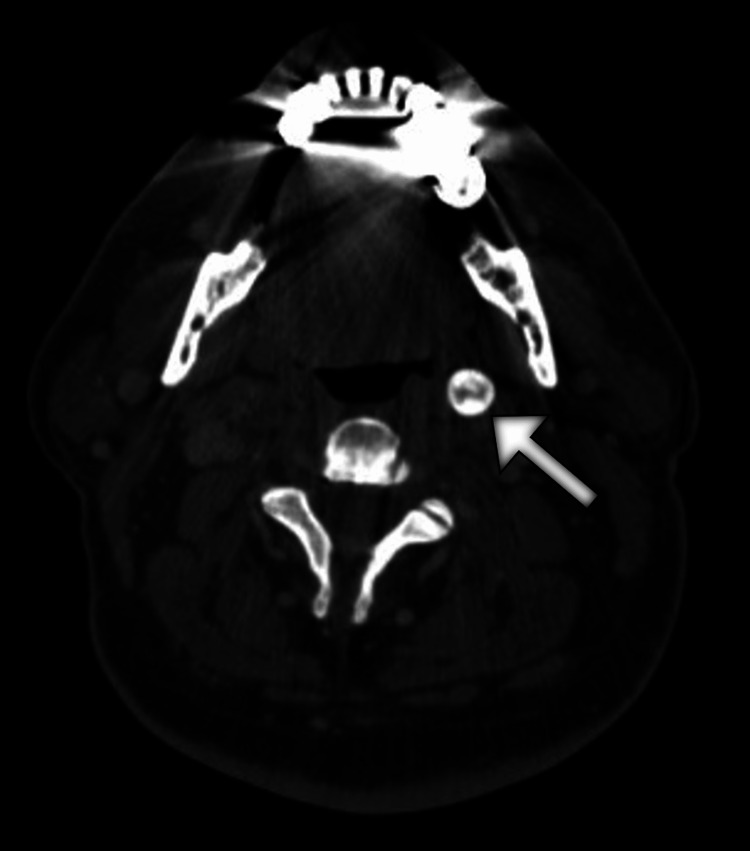
CT image of the neck shows a markedly thickened and prolonged left styloid process (arrow). CT: computed tomography

**Figure 2:**
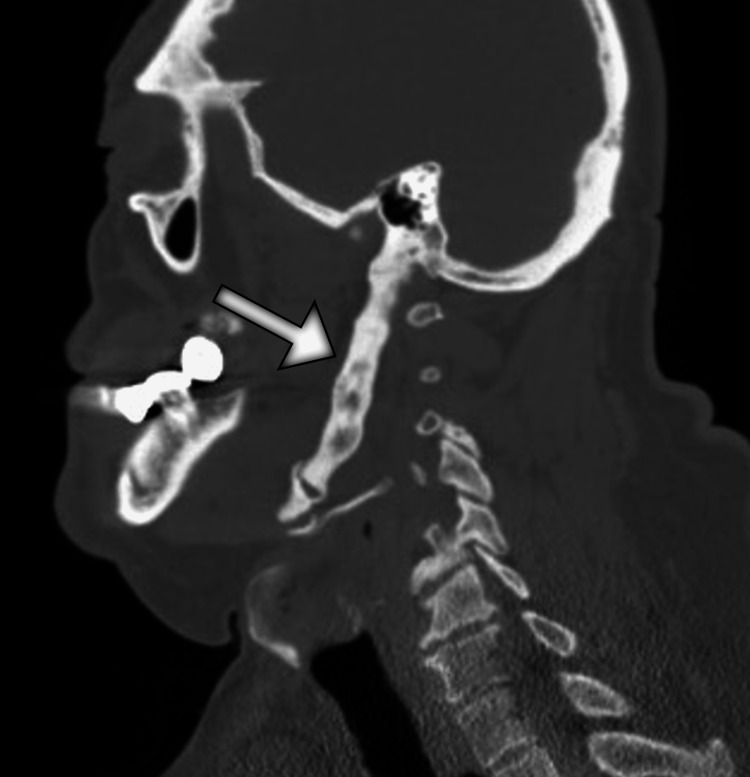
Sagittal CT image of the neck shows a markedly thickened and prolonged left styloid process (arrow) that reaches the hyoid bone. CT: computed tomography

**Figure 3:**
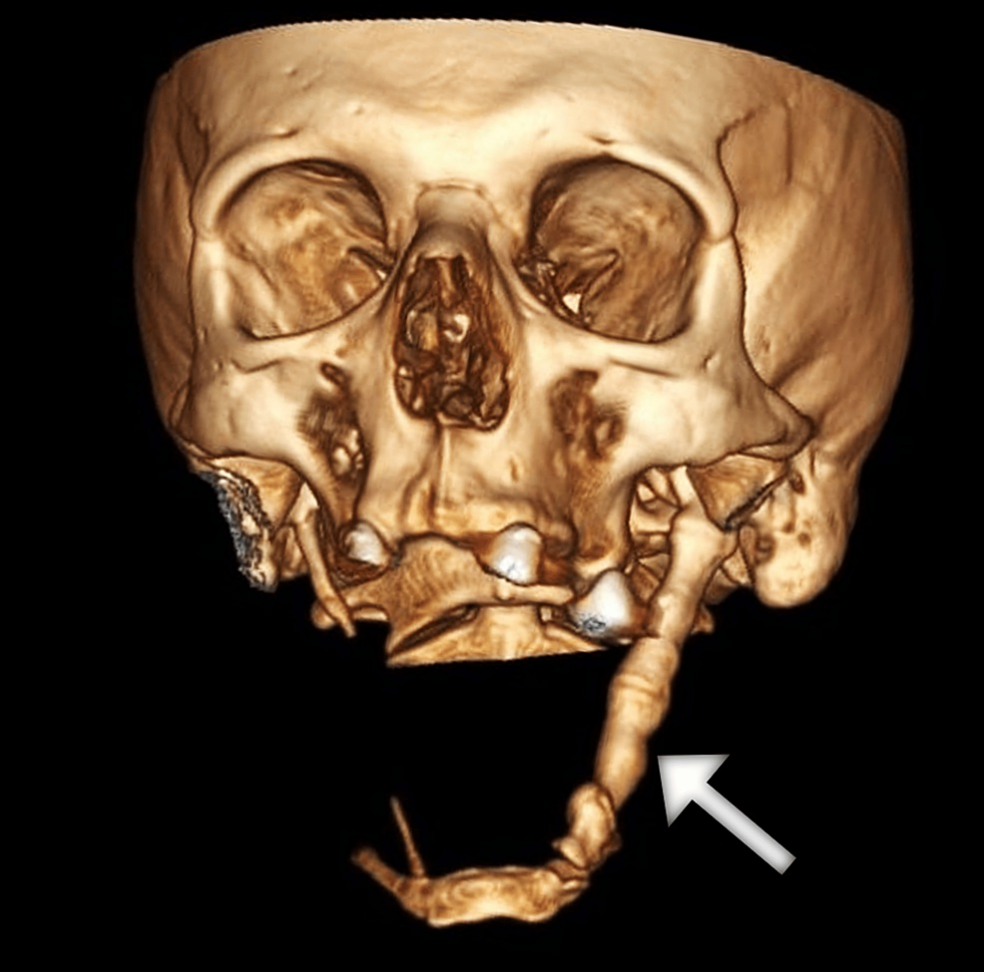
Three-dimensional CT image of the head shows a prolonged left styloid process (arrow). CT: computed tomography

Due to the progressive nature of symptoms and lack of significant improvement with the empiric treatment with a proton pump inhibitor, the patient underwent upper gastrointestinal endoscopy, which revealed a normal finding. Furthermore, the results of esophageal manometry were within normal limits. Given the recent symptoms of neck pain, the patient underwent a head and neck computed tomography (CT) scan. The scan demonstrated an extensive elongation and marked thickening of the left styloid process extending anterolaterally and caudally from the skull base to the hyoid bone and measured 14.9 cm (Figures 1-3).

The clinical and imaging findings were consistent with Eagle syndrome due to the elongation of the left styloid process. The diagnosis was discussed with the patient and the decision was made to perform a resection of the left styloid process. Careful dissection was made to separate the styloid muscles. The procedure was performed under general anesthesia and using external access approach. The patient did not have any intra-operative or postoperative complications. In particular, the functions of the muscles of the neck were intact postoperatively. The patient was discharged on the sixth postoperative day. At the one-month follow-up visit, the patient reported near-complete resolution of pain and difficulty in swallowing and had no active complaints.

## Discussion

We report a rare case of Eagle syndrome with a very long right styloid process. Eagle syndrome is a rare condition, but its reported prevalence is variable in the literature. This variation is related to the different cut-off values for the normal length of the styloid process. For example, some studies indicated that the normal length of the styloid process should not exceed 4 cm [6]. However, it should be noted that not all patients with prolonged styloid processes have Eagle syndrome because more than 4% of the normal population have a styloid process longer than 4 cm [6]. To the best of our knowledge, the current case of Eagle syndrome with a styloid process of nearly 15 cm represents the longest reported length of the styloid process in the literature. Before this, the longest styloid process causing Eagle syndrome was reported by Kubikova and Varga for a patient who had a styloid process of 14 cm bilaterally [3].

Eagle syndrome can have a myriad of clinical presentations. As in the present case, it can be a cause of dysphagia. It may also present with neck pain and globus sensation. The styloid process may result in the compression of carotid arteries, which may result in ischemic stroke or syncope [4,6,7]. Hence, it is very important to keep this diagnosis into consideration. The diagnosis can be confirmed by demonstrating the long styloid process. This can be seen in plain radiographs or panoramic radiographs. As in the present case, CT is the method of choice in diagnosis, as it shows the exact location and size of the styloid process and provides anatomical relationships of the styloid process in the anatomical neck spaces.

Surgical management is the definitive treatment of Eagle syndrome, which can be performed intraoral or external approach [3,8]. In the present case, the external cervical approach was made because of the extremely long styloid process. However, the intra-oral approach has the benefit of better cosmetic results since it does not have any external scars [3].

The exact pathophysiological mechanism for the prolonged styloid process remains unclear. It has been suggested that the prolonged styloid process in Eagle syndrome is related to retained embryonic cartilaginous tissue of Reichert cartilage. Other proposed mechanisms included ossification of the styloid ligament, development of osteitis tissue in prolapse of the styloid ligament, endocrine disorders in postmenopausal females with accompanying ossification of other ligaments, trauma, or mechanical stress against intrauterine development [4,9].

## Conclusions

Eagle syndrome is a very rare etiology of dysphagia. The case highlights an example of Eagle syndrome with an extremely long styloid process. Keeping a high index of suspicion of this condition is essential when dealing with patients with dysphagia and neck pain. The diagnosis can be easily made by imaging studies. Surgical treatment with excision of the styloid process results in a satisfactory improvement in patient symptoms.

## References

[1] Adkins C, Takakura W, Spiegel BM, Lu M, Vera-Llonch M, Williams J, Almario CV (2019). Prevalence and characteristics of dysphagia based on a population-based survey. Clin Gastroenterol Hepatol.

[2] Michaud PL, Gebril M (2021). A prolonged time to diagnosis due to misdiagnoses: a case report of an atypical presentation of eagle syndrome. Am J Case Rep.

[3] Kubikova E, Varga I (2009). A case of extremely long styloid process without clinical symptoms and complications. Clin Anat.

[4] Badhey A, Jategaonkar A, Anglin Kovacs AJ (2017). Eagle syndrome: a comprehensive review. Clin Neurol Neurosurg.

[5] Pradhan U, Adhikari TR (2022). Diagnostic and therapeutic dilemma in orofacial pain: a rare case of bilateral Eagle syndrome. SAGE Open Med Case Rep.

[6] Piagkou M, Anagnostopoulou S, Kouladouros K, Piagkos G (2009). Eagle's syndrome: a review of the literature. Clin Anat.

[7] Schoeff S, Mukherjee S (2014). Cervicogenic pain in Eagle's syndrome. Cephalalgia.

[8] Bakshi SS (2016). An unusually long process: Eagle syndrome. Am J Med.

[9] Panwar A, Keluskar V, Charantimath S, Kumar SL (2022). Bilateral elongated styloid process (eagle’s syndrome)-a case report and short review. Acta Oto-Laryngol Case Rep.

